# Prey type and foraging ecology of Sanderlings *Calidris alba* in different climate zones: are tropical areas more favourable than temperate sites?

**DOI:** 10.7717/peerj.1125

**Published:** 2015-08-11

**Authors:** Kirsten Grond, Yaa Ntiamoa-Baidu, Theunis Piersma, Jeroen Reneerkens

**Affiliations:** 1 Chair in Global Flyway Ecology, Conservation Ecology Group, Groningen Institute for Evolutionary Life Sciences (GELIFES), University of Groningen, Groningen, The Netherlands; 2 Centre for African Wetlands, University of Ghana, Accra, Ghana; 3 Department of Animal Biology and Conservation Science, University of Ghana, Accra, Ghana; 4 Department of Marine Ecology, NIOZ Royal Netherlands Institute for Sea Research, Den Burg, The Netherlands

**Keywords:** Benthic invertebrates, Shorebirds, Time budgets, Energy budgets, Differential migration, Migration

## Abstract

Sanderlings (*Calidris alba*) are long-distance migratory shorebirds with a non-breeding range that spans temperate and tropical coastal habitats. Breeding in the High Arctic combined with non-breeding seasons in the tropics necessitate long migrations, which are energetically demanding. On an annual basis, the higher energy expenditures during migration might pay off if food availability in the tropics is higher than at temperate latitudes. We compared foraging behaviour of birds at a north temperate and a tropical non-breeding site in the Netherlands and Ghana, respectively. In both cases the birds used similar habitats (open beaches), and experienced similar periods of daylight, which enabled us to compare food abundance and availability, and behavioural time budgets and food intake. During the non-breeding season, Sanderlings in the Netherlands spent 79% of their day foraging; in Ghana birds spent only 38% of the daytime period foraging and the largest proportion of their time resting (58%). The main prey item in the Netherlands was the soft-bodied polychaete *Scolelepis squamata*, while Sanderlings in Ghana fed almost exclusively on the bivalve *Donax pulchellus*, which they swallowed whole and crushed internally. Average availability of polychaete worms in the Netherlands was 7.4 g ash free dry mass (AFDM) m^−2^, which was one tenth of the 77.1 g AFDM m^−2^ estimated for the beach in Ghana. In the tropical environment of Ghana the Sanderlings combined relatively low energy requirements with high prey intake rates (1.64 mg opposed to 0.13 mg AFDM s^−1^ for Ghana and the Netherlands respectively). Although this may suggest that the Ghana beaches are the most favourable environment, processing the hard-shelled bivalve (*D. pulchellus*) which is the staple food could be costly. The large amount of daytime spent resting in Ghana may be indicative of the time needed to process the shell fragments, rather than indicate rest.

## Introduction

Shorebirds from the same breeding population are known to migrate different distances, but the proximate and ultimate factors underlying differential migration are still largely unknown (Cristol, Baker & Carbone, 1999; Piersma, 2007; Reneerkens et al., 2009). Several mechanisms have been proposed, including latitudinal differences in foraging conditions, predation risk, inter and intra-specific competition and climate. Prey burying depth and variation in bill length were shown to drive differential migration in Western Sandpipers (*Calidris mauri*), Black-tailed Godwits (*Limosa limosa*) and Bar-tailed Godwits (*Limosa lapponica*) (Mathot, Smith & Elner, 2007; Catry et al., 2012; Duijns et al., 2014). Choice of non-breeding sites in birds is also affected by latitudinal variation in predation risk (Nebel & Ydenberg, 2005; Díaz et al., 2013), as was shown for Western Sandpipers for which predator escape performance was one of the factors correlating with segregation between and within sexes at non-breeding sites (Nebel & Ydenberg, 2005).

A factor that has been shown to impact shorebird distribution on a local scale is inter- and intra-specific competition (Fernández et al., 2003; van Gils et al., 2015). Black-tailed Godwit populations at preferred breeding and non-breeding sites were observed to remain relatively stable, while steep population increases were documented in potentially less favourable areas indicating the presence of a buffer effect (Gill et al., 2001; Gunnarsson et al., 2005). A similar effect was shown for Sanderling populations in coastal Ghana, where non-breeding populations of Sanderlings (*Calidris alba*) increased in overall size, resulting in possible increased intra-specific competition of Sanderlings at preferred non-breeding sites (Ntiamoa-Baidu et al., 2014).

Sanderlings are long-distance migrants that breed in the High Arctic and have a world-wide non-breeding distribution (Reneerkens et al., 2009; Scott, 2009). Sanderling non-breeding areas span over 100° of latitude and include coastal habitats at north temperate, tropical and south temperate latitudes (Maron & Myers, 1985). Sanderlings migrating to temperate non-breeding areas have short migration distances, but are subjected to harsher climatic conditions during the non-breeding season (Castro, Myers & Ricklefs, 1992). In comparison, Sanderlings that spend the non-breeding season in tropical or south temperate areas benefit from higher ambient temperatures and more stable climatic conditions, but can be negatively affected by increased energy expenditure associated with longer migratory distances. Different stages of the avian life cycle have different energetic demands, of which migration and breeding are usually regarded the most energetically demanding stages (Drent & Piersma, 1990; Piersma, 2002; Piersma et al., 2003; Buehler & Piersma, 2008). Energy requirements are likely to differ between tropical and temperate non-breeding areas due to variation in climatic conditions and food availability. At lower ambient temperature, endotherms need to allocate more energy to thermoregulation (Wiersma & Piersma, 1994; Kersten et al., 1998). Castro, Myers & Ricklefs (1992) measured daily energy expenditure (DEE) of Sanderlings spending the non-breeding period at two north temperate and two tropical sites in the Americas and showed that DEE was almost twice as high in Sanderlings at the most northern temperate site in New Jersey, compared to a tropical site in Panama. DEE was also negatively correlated to ambient temperature which explained 70% of the variance in DEE (Castro, Myers & Ricklefs, 1992). High energy expenditures as a result of lower ambient temperatures result in higher energy requirements by Sanderlings that use temperate non-breeding sites. Therefore, selection of non-breeding area can potentially have large effects on daily energy budgets of Sanderlings, and this potentially affects their demography at the scale of the population due to different fitness parameters of individuals wintering at different locations (Alves et al., 2013a).

Food availability and quality are directly related to suitability of habitat for non-breeding shorebirds (Escudero et al., 2012; van Gils et al., 2013), as only abundant and/or high quality food can sustain shorebirds if climatic conditions are unfavourable (Ruthrauff, Gill & Tibbitts, 2013). Sanderlings have been reported to use a wide variety of food sources, including arthropods and polychaetes (Perez-Hurtado, Goss-Custard & Garcia, 1997; Petracci, 2002; Castro et al., 2009). Foraging conditions have been shown to shape migratory patterns in shorebirds (Nebel, 2007; Mathot, Smith & Elner, 2007; Catry et al., 2012; Alves et al., 2013b).

In this study we compared activity patterns and foraging behaviour of Sanderlings and prey abundance at a temperate and tropical non-breeding site. We aimed to estimate energy intake at two locations by comparing activity patterns, foraging behaviour and prey characteristics of Sanderlings, as well as ascertain how the observed energy requirements of Sanderlings compare to published estimates of energy expenditure of Sanderlings and other shorebird species. With our study, we provide insights into the importance and role of foraging conditions in the choice of non-breeding sites for shorebirds.

## Methods

### Study sites

**Figure 1:**
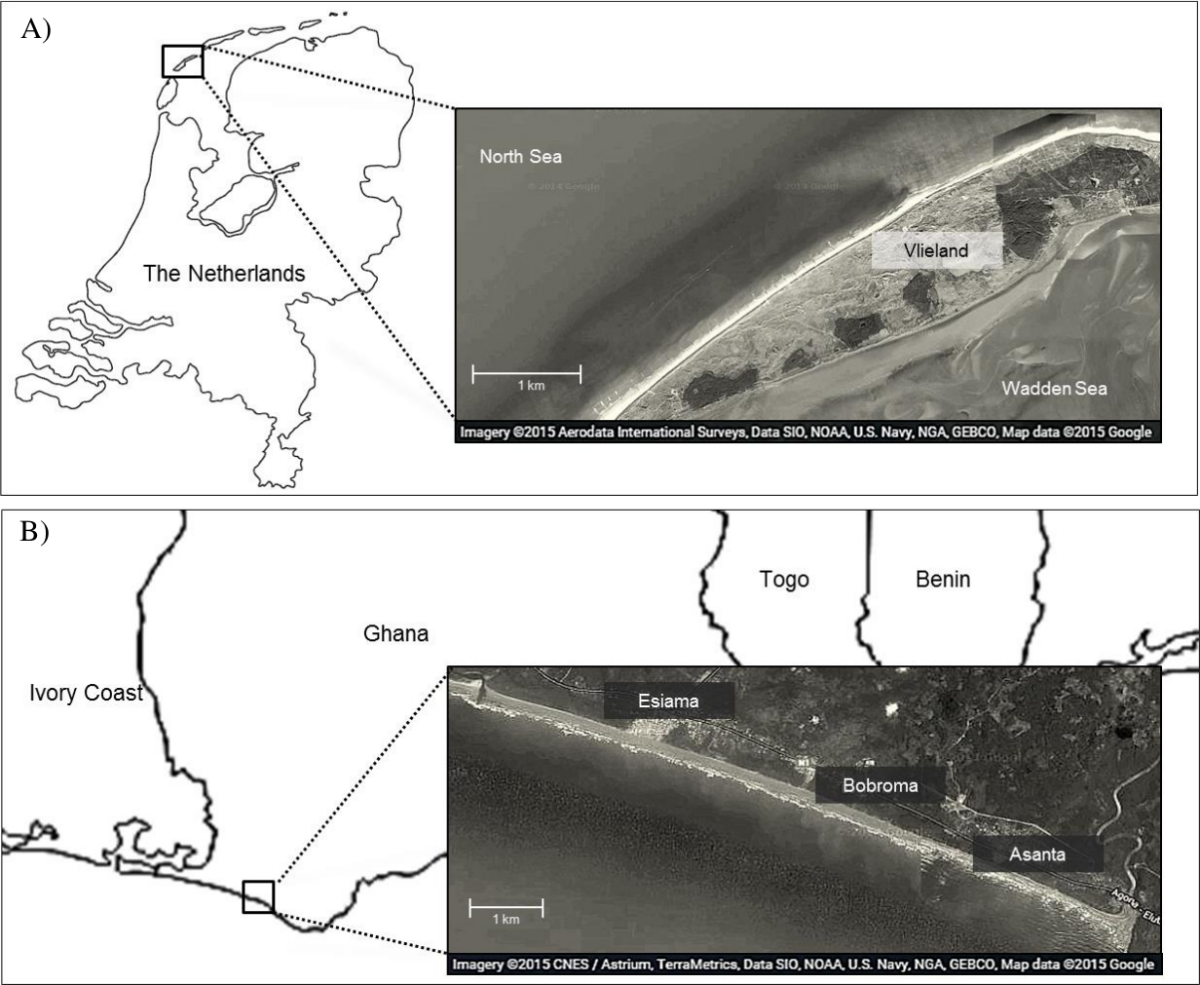
Map of study area in Vlieland, the Netherlands (A), and Esiama, Ghana (B). Satellite imagery was derived from Google and can be attributed to: Aerodata International Surveys, Data SIO, NOAA, US Navy, NGA, GEBCO, Map data 2015 (A) and CNES/Astrium, TerraMetrica, Data SIO, NOAA, US Navy, NGA, GEBCO, Map data 2015 (B).

We studied foraging ecology of Sanderlings at Vlieland, an island in the Wadden Sea in the Netherlands (53°16N, 4°55E), and at the beach of Esiama (4°56N, 2°21W), situated 300 km west of Accra, Ghana (Fig. 1). At Vlieland, Sanderlings were studied along a 6 km stretch of the North Sea beach for one season between September and November 2007. In Ghana, we studied Sanderlings for two seasons from mid-January to mid-March (2008) and from January to early February (2009). Round trip migration distances from their arctic breeding sites to non-breeding sites in the Netherlands and Ghana approximate 6,300 km and 16,000 km, respectively. Sanderlings remain at temperate non-breeding sites from mid-July to late May, and at tropical non-breeding sites from mid-August until late April (Reneerkens et al., 2009; Lemke, Bowler & Reneerkens, 2012; Ntiamoa-Baidu et al., 2014). Our study sites differed extensively in climatic and site characteristics (Table 1). The weather in the Netherlands in autumn and winter was unstable and heavy storms were not uncommon during this period. Esiama beach (Ghana) stretches over 13 km and is enclosed by the estuaries of two rivers, and the shore was lined with coconut tree plantations (Ntiamoa-Baidu et al., 2014). Weather in Esiama was stable with few heavy rain showers and little variation in ambient temperature. Day length was very similar between the Netherlands and Ghana during our study period, averaging 11.25 and 11.95 h, respectively. As night foraging is common amongst shorebirds (Robert & McNeil, 1989; Nol et al., 2014), we extrapolated all estimates of foraging time and energy intake over a 24 h period. Foraging efficiency at night may be lower for visual foragers such as Sanderlings, but we could not take this into account, as no estimates are available. We considered our two sites valid for comparison of Sanderling foraging ecology as both consisted of beach habitat (see e.g., Piersma, de Goeij & Tulp (1993) for issues of comparison).

**Table 1:** Study site characteristics of the Netherlands and Ghana.^a^

Site	Years	Months	Day length (h)	Minimum temp (°C)	Maximum temp (°C)	Tidal amplitude (m)
Vlieland, the Netherlands	2007	Sept–Nov	13.2–9.3	3–10	8–17	2.3
Esiama, Ghana	2007, 2008	Jan–Mar	11.8–12.1	23–24	30–31	1.7

**Notes.**

a Notes: Local day length for the Netherlands and Ghana was taken from www.dateandtime.info. Climate data was derived from the Royal Netherlands Meteorological Institute (www.knmi.nl), and tide data was taken from Rijkswaterstaat—Ministry of Infrastructure and the Environment (www.rijkswaterstaat.nl) for the Netherlands and from www.tide-forecast.com/locations/Takoradi-Ghana for Ghana.

We spent a total of 37 and 36 field days in the Netherlands and Ghana, respectively, covering 274 and 260 h of observation for time budget estimates. Our observations were spread out over the daylight hours, resulting in 41% of observations between 6 am and 12 noon, and 59% between 12 noon and 6 pm. To monitor prey intake, we observed Sanderlings foraging for a total of 520 and 490 min in the Netherlands and Ghana, respectively. All observations were conducted on wild Sanderlings in their natural environment and we did not handle birds for our study. Therefore, no permission from the Animal Ethics Committee of the University of Groningen (DEC-RUG) was required.

### Invertebrate sampling

We studied the marine benthic invertebrate community to assess variation and availability of different prey species for Sanderlings. In September/October 2008 (Netherlands) and January 2009/2010 (Ghana), we collected a total of 181 and 614 samples of benthic invertebrates in the upper 20 cm of marine sediment, using sediment cores with inner diameters of 14.9 cm (0.0174 m^2^) and 12.5 cm (0.0123 m^2^). We collected benthos at 100 m intervals at the tide line, where Sanderlings forage. In 2008, we sampled over the whole length of both study areas seven times during different stages of the tidal cycle, using the tidal variation to obtain samples at different elevations. We also sampled the Ghanaian study area twice in 2010 to examine inter-annual variation in the benthic invertebrate community. We sieved samples over 1 mm mesh size on site and benthic invertebrates were frozen until further analyses. We extrapolated benthic invertebrate densities and energetic value to m^2^.

To assess invertebrate availability to Sanderlings, we measured burying depths of 300 shellfish in Ghana by extracting a core and systematically scraping off vertical layers of sediment. Depth of shellfish exposed was measured from the top of the core to the middle of the shell. We were unable to measure burying depths of the polychaetes in the Netherlands, because they responded to our presence by quickly retreating deep into the sediment.

Benthic invertebrates were identified to species level, counted and measured to the nearest mm. We dried invertebrate samples at 60 °C for two days, determined ash-free dry mass (AFDM) by subtracting ash weight from dried sample weight and determined ash mass using the end product of processing the samples in a bomb-calorimeter. We used a bomb-calorimeter to estimate energy content of our polychaete and other invertebrate samples. Where possible, samples were processed as duplicates to assess measurement precision, and values were averaged. For shellfish, we used a fixed value of 22 kJ g^−1^ AFDM, in further analyses due to inconsistencies in energy measurements (Zwarts & Wanink, 1993). We calculated prey quality following Yang et al. (2013), using:

\begin{eqnarray*} Q=d\times a\times \frac{\mathrm{AFDMflesh}}{\mathrm{DMshell}} \end{eqnarray*}

where *d* is the energetic density of the flesh (22 kJ g^−1^ AFDM) and *a* is digestion efficiency (0.82) (Castro et al., 2008; Yang et al., 2013). Size differences between shells were not taken into account, as the majority (>90%) of shells collected measured between 0.5 and 0.7 cm.

### Time budgets

We conducted daily surveys covering the entire study area and recorded time, place and behavior of each individual encountered. We categorized time of observations into hours before and after low tide, enabling us to relate behavior to the tidal cycle. Our ethogram included: (1) Foraging: birds were actively foraging and ingesting food; foraging included both probing and pecking, (2) Resting: birds were not foraging, but stood or lied down with little or no apparent movement, (3) Preening: birds actively preened and/or bathed, and (4) Locomotion: birds were actively moving between locations. Locomotion included all running and flying between foraging and roosting sites. Based on the time birds spent exerting different behaviors over the period of each survey, we calculated daily time budgets of Sanderlings at both study sites.

### Prey intake

We observed individual Sanderlings for up to 10 min through a spotting telescope to determine food intake rate. To ensure independence of observations among foraging birds, we focused our observations on color-banded individuals and avoided recording measurements from the same individuals within the same flock on the same day. We measured prey intake rate, and identified prey type when possible. We recorded successful ingestion of large-bodied prey by counting swallowing movements of the foraging bird, identified by a fast upward movement of the head. Energy intake was calculated as the weighted average of intake rates observed from Sanderlings foraging during the whole tidal cycle and extrapolated to energy intake/hour, using known energy contents of prey items. Due to distance and the high foraging speed of Sanderlings, prey could only be classified as polychaete or non-polychaete during field observations. We calculated prey intake rates by multiplying the energy values we determined for polychaete and non-polychaete prey by the observed prey ingested during timed foraging observations. For polychaete prey, energetic content estimated we included polychaetes >20 mm in size, as we were unable to observe ingestion of smaller polychaetes. We did not differentiate between polychaetes >20 mm in our calculations, as we could not accurately estimate prey size in the field.

As a gauge for the energy requirements at the two sites, we used the empirical value for DEE measured in Texas in winter (135 kJ/day) by Castro, Myers & Ricklefs (1992) for the Netherlands. Ambient temperature during the Texas winter was comparable to average autumn temperatures in the Netherlands during our study (10 °C and 14 °C, respectively). As an approximation of DEE in Ghana we used 100 kJ/day, which was measured for Sanderlings spending the non-breeding season in Panama. Temperatures between Panama and Ghana are also comparable (27 °C and 32 °C on average) and are above the lower critical temperature of 23 °C estimated for Sanderlings (Kersten et al., 1998).

### Statistics

We used a Generalized Linear Model to compare proportion of foraging Sanderlings between sites and at different times of the tidal cycle. Our full model is shown below:

\begin{eqnarray*} \% \mathrm{Foraging}\sim \mathrm{Site}+\mathrm{Tide}+{\mathrm{Tide}}^{2}+\mathrm{Site}\ast \mathrm{Tide}+\mathrm{Site}\ast {\mathrm{Tide}}^{2} \end{eqnarray*}

Tide was expressed in hours before and after low tide. We used Tide^2^ because we expected a quadratic relationship between proportion of birds foraging and time in the tidal cycle. All statistical analyses were conducted using the R environment for statistical programming version 3.1.0 (R Development Core Team, 2010). All means are presented ±1SE.

## Results

### Food availability and quality

**Figure 2:**
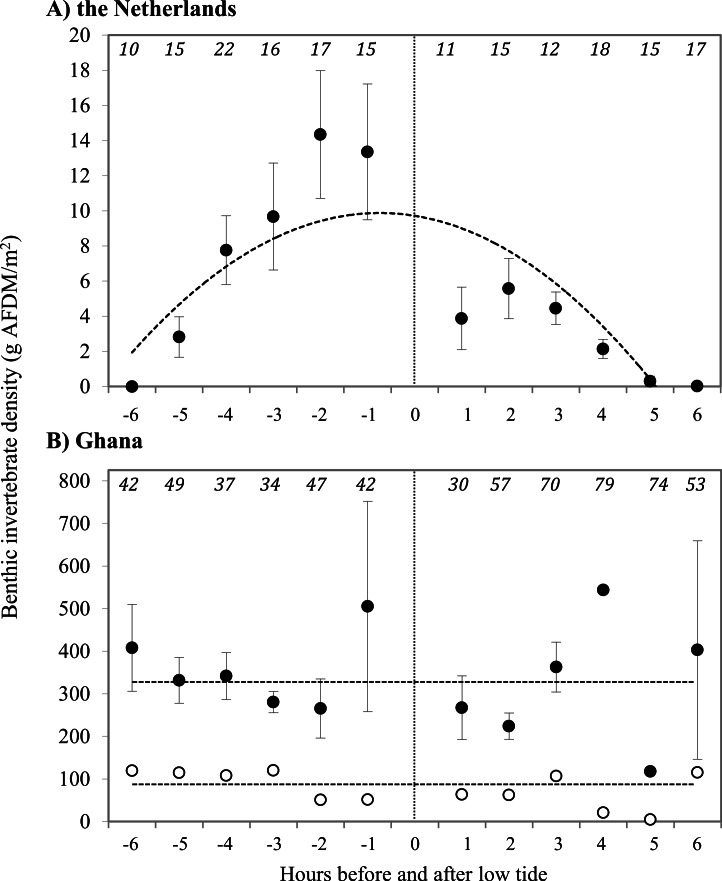
Density of prey at the tide line in g **AFDM/m^2^** at (A) Vlieland, the Netherlands, and (B) Esiama, Ghana. Invertebrate densities in Ghana are shown as average values of all samples (open circles) and average values of samples collected only from the shell bank (closed circles). (A) *Y* = − 6.086*x*^2^ + 71.207*x* − 35.117, *R*^2^ = 0.6414 (*t* = − 3.825, *p* = 0.004). (B) Dashed lines represent the average benthic invertebrate densities, as correlations were not significant. Open: 337.4 g AFDM/m^2^ (*t* = − 0.900, *p* = 0.3914); Closed: 78.1 g AFDM/m^2^ (*t* = 0.303, *p* = 0.768). The *x*-axis represents hours before and after low tide, with low tide (0) marked by the dotted line. −6 h before low tide corresponds to high tide, with water receding towards low tide at (0). 0–6 h represent the incoming tide from low (0) to high (6). Number of sediment cores collected are shown in italics at the top of the graphs. Error bars represent standard error.

Prey type, abundance and energetic content differed extensively between sites. In the Netherlands, 94.5% of the benthic species in our samples consisted of the polychaete *Scolelepis squamata*, with the amphipod *Haustorius arenarius* (4.5%) as the second most abundant prey species. The biomass of sanderling prey at the beach in the Netherlands was 7.4 ± 1.6 g AFDM m^−2^, and invertebrate distributions were positively correlated with the timing relative to low tide (Fig. 2). The average energy values of these two invertebrate species were 40.5 ± 0.41 kJ g^−1^ and 20.3 ± 0.04 kJ g^−1^ AFDM, respectively. For AFDM in the Netherlands, time within the tidal cycle explained 64% of the variation in biomass (*R*^2^ = 0.64; Fig. 2A). The highest densities of *S. squamata* were found furthest away from the high tide line, and thus only available during low tide. Average AFDM g^−1^ sample for polychaetes of different sizes (20 mm–>50 mm) varied between 0.75 and 0.88, with an overall average of 0.83 ± 0.01 g AFDM g^−1^ sample. These intake rates are only based on the ingestion of polychaetes, as we were unable to identify non-polychaete prey in the field. Average polychaete prey capture rate was 0.075 polychaete s^−1^ during our 10 min intake observations. The benthic fauna of the Ghana beach was dominated by the bivalve *Donax pulchellus* which accounted for over 95% in numbers of all organisms found in the samples. Sanderlings were observed to feed almost exclusively on this bivalve. *Hydrobia ulvae* was the second most abundant species in Ghana with highest densities further away from the shore than *D. pulchellus*. *D. pulchellus* were distributed over the whole width of the beach with an average energetic content of 77 g AFDM m^−2^, but occurred in highest densities in a 1–3 m wide shell bank (up to 170,000 individuals m^−2^/1,146 g AFDM m^−2^), that was present in both 2008 and 2009 and clearly visible. Although we sampled benthos up to a depth of 20 cm, we only detected invertebrates in the top sediment layer. *D. pulchellus* had a burying depth of 1.09 ± 0.72 cm, and were thus always available to the Sanderlings, which have bill lengths of 2.5 cm. Sanderlings in the Netherlands were observed to feed on *S. squamata* and small prey unidentifiable by sight by us. Polychaetes accounted for 45% of successful prey intakes, and the other 55% was composed of small, unidentifiable prey items. Prey quality of *D. pulchellus* was estimated at 4.59 ± 0.12 kJ g^−1^ DM_shell_, and quality of *S. squamata* and *H. arenarius* were 110.6 ± 1.9 and 82.6 kJ g^−1^ DM_shell_, respectively. We processed all *H. arenarius* in a single sample, thus cannot provide a SE estimate.

### Sanderling time budgets and prey intake

**Figure 3:**
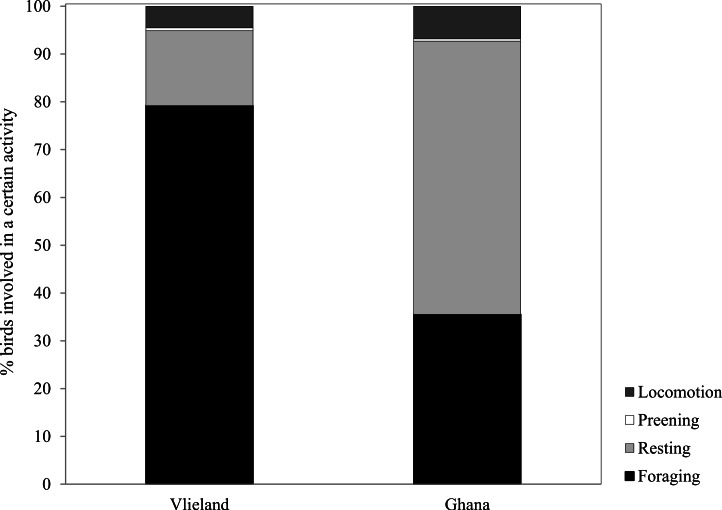
Percentage of time spent by Sanderlings in the Netherlands (Vlieland) and Ghana (Esiama) on the four major behaviours observed.

**Figure 4:**
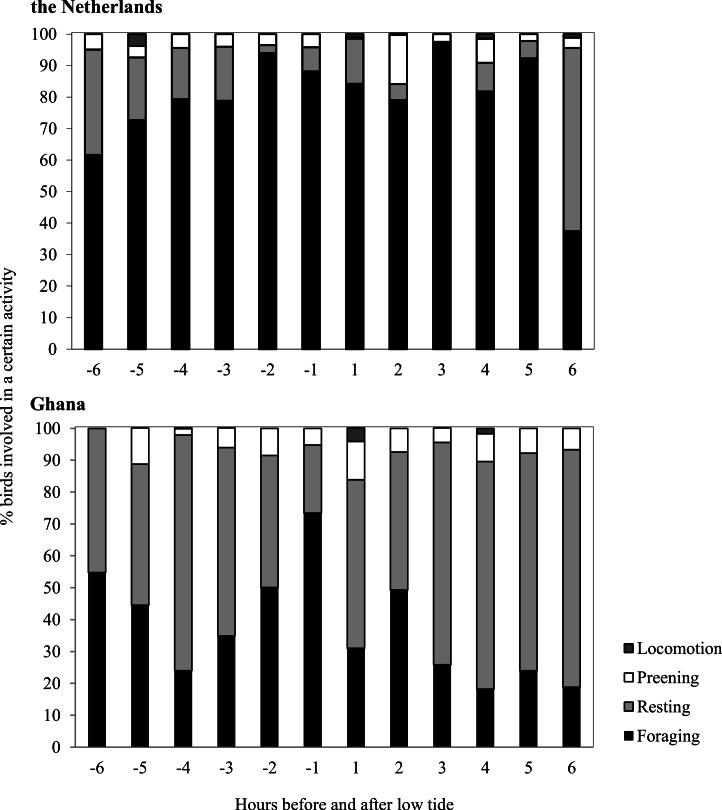
Time budgets of Sanderlings in the Netherlands and Ghana classified per hour before and after low tide. Zero on the *x*-axis represents low tide and 6 and −6 represents high tide.

Average Sanderling densities were 34 ± 0.7 birds km^−1^ in the Netherlands and 214 ± 2.6 birds km^−1^ in Ghana, respectively. We estimated density over a one-dimensional space that covered the length of the beach. In the Netherlands, Sanderlings were spread out on the beach or in small groups (<10 birds) while in Ghana they occurred in large flocks (>50 birds). Time budgets of Sanderlings during the non-breeding period in the Netherlands and Ghana differed predominantly in time spent foraging and resting (Fig. 3). We adjusted time budget calculations to average day lengths in the Netherlands and Ghana during our study period. In the Netherlands, the Sanderlings’ main activity was foraging, which comprised 68.3% (17.8 h) of their time, while in Ghana, Sanderlings foraged 35.1% (9.0 h) of the time. In Ghana, 56.4% of the birds observed were resting, opposed to 13.6% in the Netherlands. The interaction Site*Tide and Site*Tide^2^ in our model were not significant and the model we used for our analyses only included Site and Tide^2^. The proportion of Sanderlings observed to be foraging differed significantly between sites (GLM, *t*_23_ = 4.0, *p* < 0.0007), but no significant correlation between the proportion of foraging birds and the stage in the tidal cycle was found at either site (GLM, *t*_23_ = − 0.576, *p* = 0.57). Preening and locomotion accounted for 4.0–8.1% and 0.5–14.1% of daily effort in the Netherlands and Ghana. Foraging and resting was independent of the tidal cycle in Ghana, but in the Netherlands the percentage of foraging birds appeared to show a slight increase around low tide, though not significant (Fig. 4).

**Figure 5:**
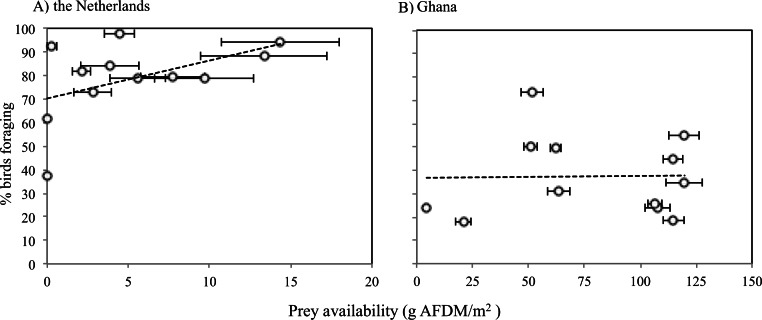
Proportions of Sanderlings foraging at different benthic invertebrate availabilities (g **AFDM/m^2^**) in the Netherlands (A) and Ghana (B). (A) *Y* = 1.6114*x* + 70.255, *R*^2^ = 0.24048. (B) average: 37.5 g AFDM/m^2^. Horizontal error bars represent standard errors.

The average caloric intake of Sanderlings was estimated to be 0.13 mg AFDM s^−1^ (2.28 J s^−1^) in the Netherlands compared to 1.64 mg AFDM s^−1^ (36.27 J s^−1^) in Ghana. For Sanderlings in the Netherlands there was a tendency for a positive correlation between foraging time and benthos availability (ANOVA, F_2_ = 3.166, *p* = 0.105), while in Ghana proportion of foraging birds was independent of the benthos availability (ANOVA, F_2_ = 0.004, *p* = 0.951) (Fig. 5).

## Discussion

Selection of non-breeding sites had a large effect on time budgets and behaviour of Sanderlings. Sanderlings in the Netherlands foraged almost twice as long as in Ghana during the non-breeding season. If tropical habitats are more energetically favourable for non-breeding Sanderlings, why do Sanderlings have such a wide non-breeding range? In tropical areas, we assumed that DEE was lower due to lower thermoregulatory costs as described by Castro, Myers & Ricklefs (1992). If our sites were comparable to sites in Castro, Myers & Ricklefs (1992) with respect to DEE, our estimated energy intake would need Sanderlings to forage 20.1 and 0.9 h per 24 h in the Netherlands and Ghana, respectively, to meet their energy requirements. For our calculations, we assumed a digestive efficiency of 82% which was estimated for Snowy Plovers (*Charadrius alexandrinus*) (Castro et al., 2008). However, if we extrapolate our observed percentages of birds foraging to a 24 h period, we find that Sanderlings in the Netherlands and Ghana forage for 19.0 and 9.0 h, respectively. Expected foraging time for the Netherlands is close to the observed foraging time, but Sanderlings in Ghana exceed expected foraging time by almost 10-fold. Food availability and quality could cause this discrepancy, because Sanderlings in two other tropical locations with similar ambient temperatures within Sanderlings’ thermoneutral zone, foraged for different periods per day. Sanderlings in Kenya were observed to spend 51%/12.4 h of their time foraging during the non-breeding period (Fasola & Biddau, 1997), which is longer than at tropical sites investigated in our study (35%/9.0 h) and by Castro, Myers & Ricklefs (1992) in Panama (40%/9.0 h).

We were only able to identify prey type captured in 45% of intake observations in the Netherlands. Our benthic samples contained amphipods such as *H. arenarius*, but abundances were too low to account for over half of our observed ingestions. A recent study identified biofilm on mud flats as an important food source for Western Sandpipers, comprising 50% of their daily energy intake (Kuwae et al., 2008; Jardine et al., 2015). Although biofilm formation occurs on some beaches as well (Piggot et al., 2012) and Sanderlings possess the tongue spine structures associated with biofilm feeding (Kuwae et al., 2012), the powerful waves at Vlieland are likely to inhibit formation of biofilm in high abundances.

Sanderlings utilize a wide variety of food sources throughout the year, which mainly consist of soft-bodied prey items. Benthic fauna in Ghana was dominated by the bivalve *Donax pulchellus*, and prey availability in Ghana was high, which could present an advantage to Sanderlings spending the non-breeding season at this site. Piersma, de Goeij & Tulp (1993) estimated that total biomass of temperate and tropical intertidal foraging areas ranged between 5 and 80 g AFDM m^−2^, with no latitudinal trend present. The average biomass at our study sites was 77 g AFDM m^−2^, which is on the richer side of the spectrum. Local biomass within the shell bank exceeded 1 kg AFDM m^−2^. The energetic benefit of bivalve prey could be lower than expected, as different prey types would require different processing costs. Red Knots develop a muscular gizzard to process food with hard indigestible shell material (Piersma, Koolhaas & Dekinga, 1993). However, after initial adaptations of the gastro-intestinal tract, no difference in energy expenditure was measured between Red Knots which were fed either soft-bodied prey or shellfish (Piersma et al., 2003). Nonetheless, reliance on a prey source that mainly consists of hard shell throughout the non-breeding season was less energetically favourable for Sanderlings than soft-bodied prey.

High processing costs and relatively low energetic gain of a bivalve diet of Sanderlings in Ghana translated into low prey quality. Prey quality of *D. pulchellus* was estimated at 4.59 ± 0.12 kJ g^−1^ DM_shell_, which was over 20 times lower than quality of the main soft-bodied prey in the Netherlands, *S. squamata*. Although low compared to soft-bodied prey, our estimate of bivalve prey quality was higher than found for Red Knot bivalve prey which ranged from 0.89 ± 0.07 kJ g^−1^ DM_shell_ in Australia (van Gils et al., 2005) to 3.50 ± 0.23 kJ g^−1^ DM_shell_ in Argentina (González, Piersma & Verkuil, 2015). Yang et al. (2013) discovered that despite low prey quality of 1.32 kJ g^−1^ DM_shell_, Red Knots were successful in pre-migratory fattening due to high food availability and local bivalves being easily crushed in the gizzard. *D. pulchellus* in Ghana are relatively large (∼7 mm) and realized prey quality could be lower to Sanderlings if hard to crush, as we based prey quality only on flesh to shell ratios and did not take crushability into account (van Gils et al., 2005). We extrapolated hourly intake from our 10 min field observations, and it is likely that the birds did not forage at this rate continuously, but either reduced prey intake or interrupted foraging bouts by short periods of rest. The latter behavior was observed in Red Knots, for which these resting periods were found to be digestive breaks. During digestive breaks, Red Knots processed ingested shell matter which refrained them from foraging at optimal rate (van Gils et al., 2004). This is likely also the case for Sanderlings if crushing and processing of *D. pulchellus* is demanding with respect to time and energetic cost. One factor that we did not take into account was the effect of differences in salinity between the Netherlands and Ghana. Tropical environments are usually characterized by high salinity (Talley, 2002), which in shorebirds can result in physiological stress such as an increased Basal Metabolic Rate (BMR) and suboptimal food intake (Gutiérrez et al., 2015). Although this can be an important factor affecting foraging ecology, our site in Ghana was flanked by two riverine estuaries, which likely decrease salinity on a local scale.

Another reason for spending the non-breeding season in the temperate zone is that the advantages of lower energy expenditure potentially may not compensate for the disadvantages of a longer migration if paired with survival costs. The survival repercussions of longer migration can be large (e.g., Sillett & Holmes, 2002; Lok, Overdijk & Piersma, 2015). In several species of resident and migratory sparrows, survival during the non-breeding season at southern sites was found to be higher, but annual survival was equal in northern non-breeding birds (Sandercock & Jaramillo, 2002).

We showed that selection of non-breeding sites can result in large differences in foraging conditions, and that Sanderlings at our tropical site encountered higher food availability but lower prey quality. Nevertheless, energy intake at our tropical site was high, which raises the question of which other ecological and physiological factors affect Sanderlings during the non-breeding season. So far, studies on Sanderling ecology have either investigated energy expenditure or energy requirements. Simultaneous assessment of energy expenditure of Sanderlings throughout their non-breeding range, diurnal and especially nocturnal foraging behaviour, and prey characteristics and availability could further elucidate the relationship between energy expenditure and requirements.

## Supplemental Information

10.7717/peerj.1125/supp-1 Supplemental Information 1 Raw data
